# Late coronary artery injury following chemoradiotherapy for thymic carcinoma: a case report

**DOI:** 10.1186/s12872-024-03948-2

**Published:** 2024-05-22

**Authors:** Sigan Hu, Jun Wang, Zhen Cui, Yongchun Zhou, Dasheng Gao

**Affiliations:** 1 Department of Cardiology, First Affiliated Hospital of Bengbu Medical University, Bengbu, 233004 China; 2 Department of Radiation Oncology, First Affiliated Hospital of Bengbu Medical University, Bengbu, 233004 China

**Keywords:** Thymic carcinoma, Acute coronary syndrome, Intravascular ultrasound, Negative remodeling

## Abstract

**Introduction:**

Surgery remains the primary treatment modality for thymic carcinoma, with adjuvant radiotherapy being recommended to effectively mitigate local recurrence and metastasis rates subsequent to incomplete or complete resection. Chemoradiotherapy has the potential to induce coronary artery occlusion, thereby potentially impacting patients’ long-term survival rates. The existing literature currently lacks comprehensive research on the lesion characteristics of coronary artery injury resulting from chemoradiotherapy.

**Case presentation:**

The male patient, aged 55, was admitted to the hospital due to recurrent chest tightness and pain persisting for one week. Notably, the patient had previously undergone curative resection surgery for thymic carcinoma seven years ago. After the surgical procedure, the patient underwent a course of adjuvant chemotherapy comprising docetaxel and platinum. 11 months later, imaging examination diagnosed tumor recurrence, and concurrent chemoradiotherapy was administered at a total dose of 62 Gy/31F for planning gross target volume (PGTV) and 54 Gy/31F for planning target volume (PTV) with 2 cycles of paclitaxel and cisplatin. Re-admission of the patient occurred after a 7-year interval subsequent to the completion of concurrent chemoradiotherapy, leading to a subsequent diagnosis of acute non-ST segment elevation myocardial infarction. Following administration of antiplatelet, anticoagulant, and anti-myocardial ischemia therapy, coronary angiography revealed the presence of a bifurcation lesion at the distal end of the left main trunk. Intravascular ultrasound (IVUS) examination demonstrated significant negative remodeling of both the main trunk and its branches at the bifurcation site, characterized by minimal atherosclerotic plaque components.

**Conclusions:**

Chemoradiotherapy may induce damage to endothelial cells, resulting in an inflammatory response. Negative remodeling of blood vessels is likely to occur, primarily characterized by vasoconstriction but with less atherosclerotic plaque burden. Routine stent implantation in negatively remodeled areas may lead to vascular rupture, necessitating intravascular imaging examination.

**Supplementary Information:**

The online version contains supplementary material available at 10.1186/s12872-024-03948-2.

## Introduction

Thymic carcinoma, a rare malignant tumor of the mediastinum (with an incidence rate of 1.5 cases per million), presents with advanced or recurrent tumors in approximately 30% of patients [1]. While surgical intervention remains the primary treatment for thymic carcinoma, adjuvant radiotherapy is also recommended to mitigate local recurrence and metastasis risks following incomplete or complete resection. However, it is worth noting that vascular damage has been reported in the literature following radiotherapy, and the potential occlusive effect of radiotherapy on coronary arteries, primarily hastening the progression of atherosclerosis [2, 3]. Currently, there is a paucity of comprehensive research in the existing literature regarding the radiological characteristics associated with coronary artery injury resulting from radiotherapy. In this study, we present a case report of a patient who underwent complete thymectomy for thymic carcinoma and subsequently received adjutant radiotherapy. The patient later presented with acute coronary syndrome necessitating hospitalization. To investigate the radiological features of coronary artery damage following radiotherapy, both coronary angiography and intravascular ultrasound(IVUS) examination were performed, providing valuable insights for clinical management.

## Case presentation

**Fig. 1:**
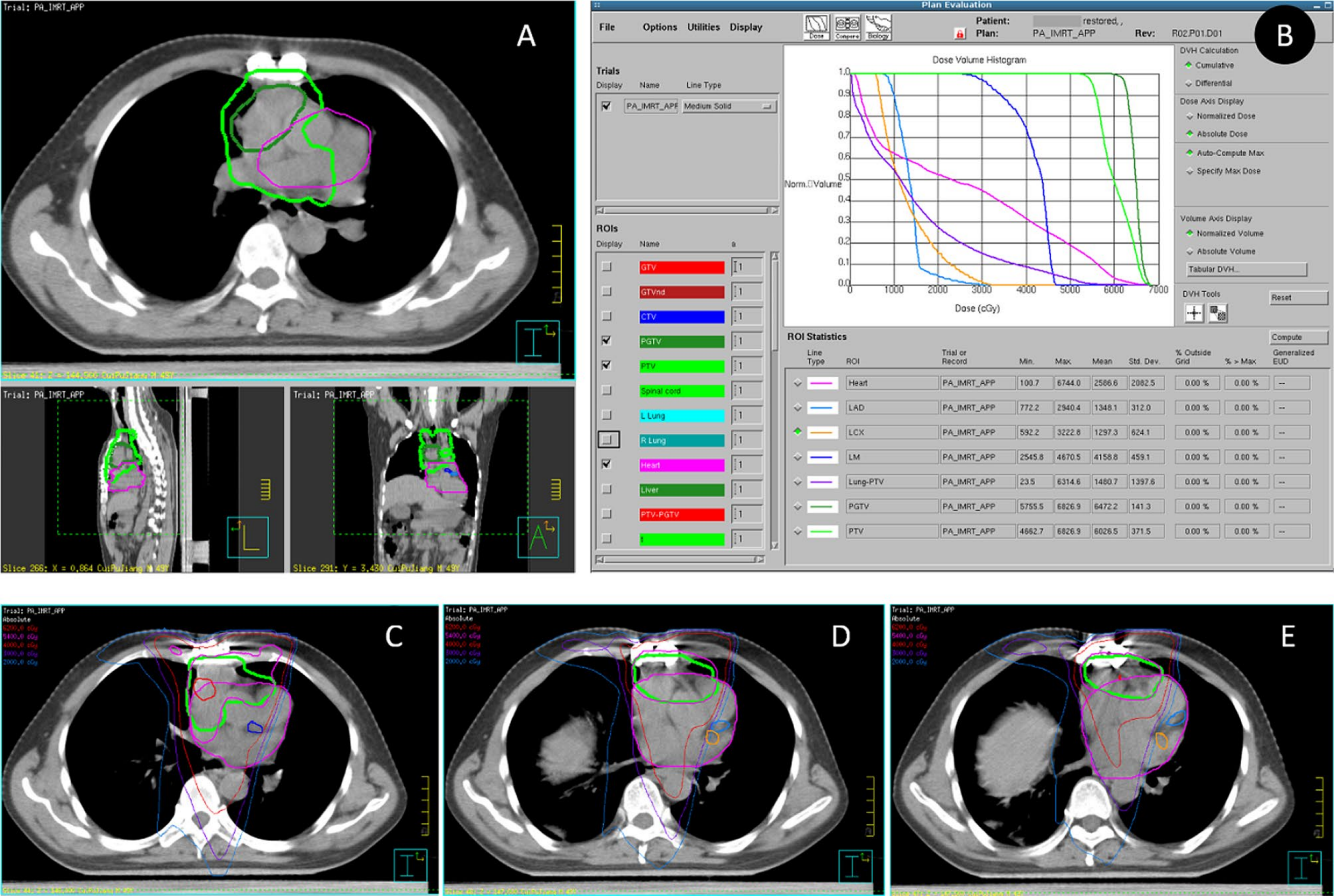
The location of recurrent tumor and the dose distribution in radiotherapy plan. The abbreviation LM refers to the left main coronary artery, which is visually represented by a distinct deep blue label. LAD stands for the left anterior descending branch of the coronary artery, depicted with a subtle light blue color. LCX signifies the left circumflex branch of the coronary artery, indicated by a prominent deep yellow hue. **A**. The location of recurrent tumor; **B.** Dose and volume histogram (DVH) of radiotherapy plan; **C**, **D** and **E**. Dose distribution of radiotherapy for different areas of coronary artery

A 55-year-old male patient was admitted to the hospital due to the presence of intermittent chest tightness and pain for one week. The patient underwent radical thymic carcinoma resection surgery 7 years ago. Intraoperatively, the tumor was identified within the thymus measuring approximately 8.0 centimeters in diameter. It exhibited a firm consistency and an incomplete capsule, infiltrating both sides of the pericardium and encasing the right upper lobe of the lung as well as surrounding the superior vena cava. The pathological diagnosis indicates the presence of thymic carcinoma. The immunohistochemical analysis revealed that the tumor cells exhibited TTF-1(-), CK7(-), CK5/6(+), CD5(+), TDT(-), P40(+), CK19(+), Ki67(+,approximately 60%). After the surgical procedure, the patient underwent 6 cycles of adjuvant chemotherapy consisting of docetaxel and platinum. 11 months later, the chest CT scan review revealed tumor was recurrent in anterior mediastinum, and intensity modulated radiotherapy (IMRT) was administered at a total dose of 62 Gy/31F for planning gross target volume (PGTV) and 54 Gy/31F for planning target volume (PTV) (Fig. 1A). The critical surrounding organs, such as the spinal cord, lungs and Heart, were subjected to stringent dosage constraints (Fig. 1B). The coronary artery were contoured by later supplementation, and the stenotic area of coronary artery were located in the irradiated field with relative high dose (Fig. 1C-E). 2 cycles of concurrent chemotherapy of paclitaxel and cisplatin regimen were combined with radiotherapy. Subsequently, the tumor condition was alleviated without any subsequent follow-up conducted.

**Fig. 2:**
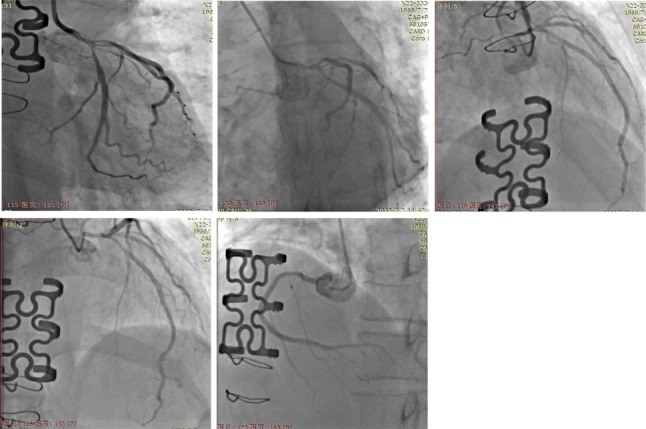
The coronary angiography findings indicate the presence of a genuine bifurcation lesion located at the distal segment of the left main coronary artery (Medina classification 1.1.1 type, with stenosis percentages of 80%/85%/80%)

**Fig. 3:**
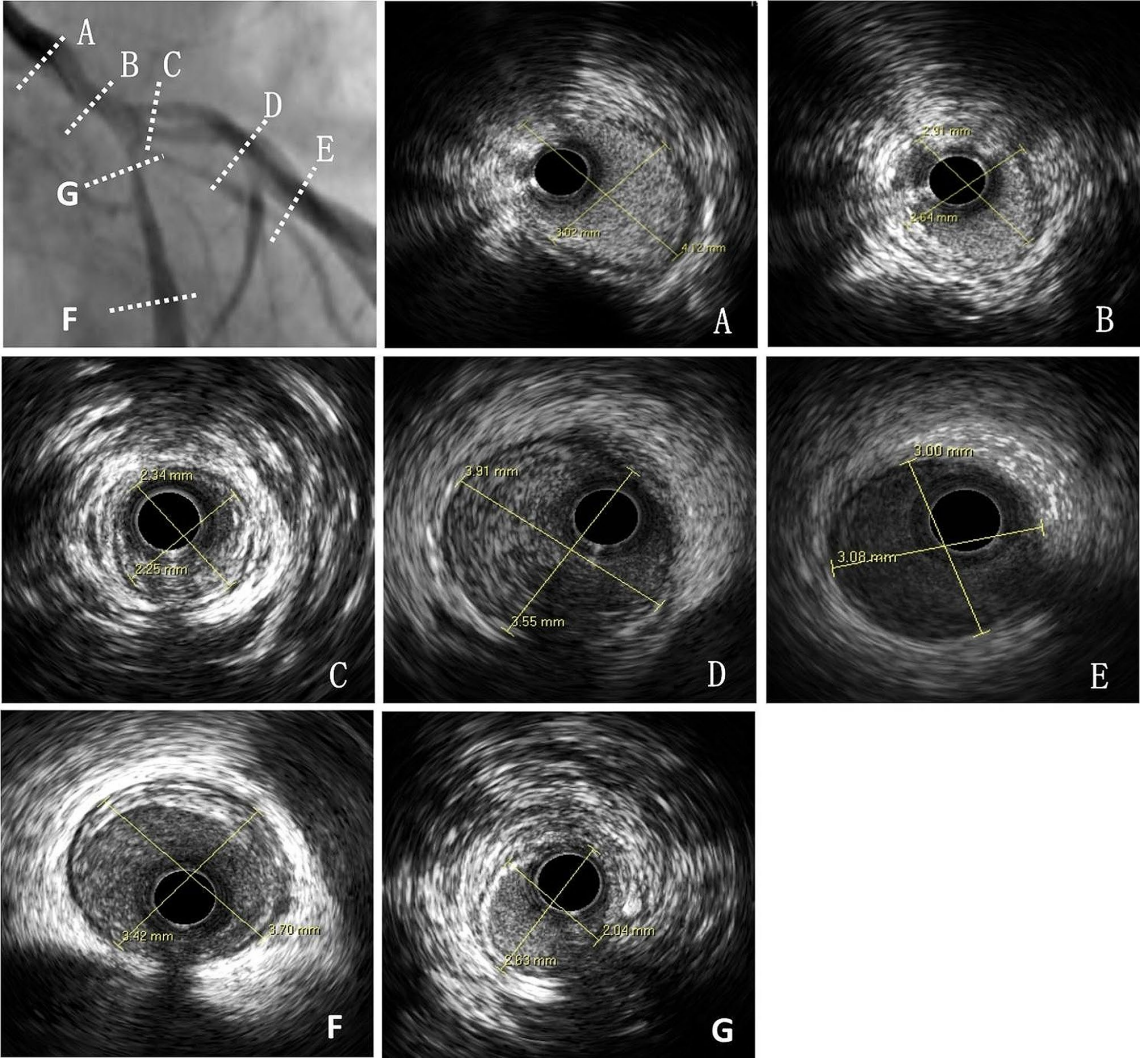
Coronary artery lesions in different segments as depicted by IVUS images: **A** indicates a proximal segment diameter of 3.02 / 4.12 mm for the left main trunk; **B** reveals a distal segment diameter of 2.64 / 2.91 mm for the left main trunk, exhibiting evident negative remodeling and minimal plaque formation; **C** demonstrates an opening to proximal diameter of 2.25 / 2.34 mm for the anterior descending branch, accompanied by significant negative remodeling and minimal fibrous plaques; **D** displays a proximal segment diameter of 3.55 / 3.91 mm for the anterior descending branch with fibrous plaque formation; **E** suggests a middle to distal diameter of 3.00 / 3.08 mm for the anterior descending branch; **F** presents a near-middle segment diameter of 3.42 /3.70 mm for the circumflex branch with some fibrous plaque formation; **G** shows an opening diameter of 2.04/2.63 mm for the circumflex branch indicating clear negative remodeling without any evidence of atherosclerotic plaques

The patient presented with recurrent chest tightness and pain for one week upon readmission without any comorbidities including hypertension, diabetes, smoking or other established risk factors associated with atherosclerosis evident from medical history review. The physical examination findings included a resting pulse rate of 64 beats per minute and blood pressure measurements of 106/71 mmHg (1 mmHg = 0.133 kPa), along with the absence of any positive clinical indicators. No abnormalities were observed on the resting electrocardiogram. However, during the episode of chest pain, the electrocardiogram demonstrated ST segment depression measuring at -0.1 mV in leads V_1 − 5_. Elevated levels were noted in creatine kinase-MB (CK-MB) at 43U/L (normal range: 0-25U/L), and cardiac troponin I (cTnI) at 0.45ug/L (normal range: < 0.03ug /L). Consequently, the diagnosis rendered was acute non-ST segment elevation myocardial infarction(NSTEMI). After administration of antiplatelet, anticoagulant, and anti-myocardial ischemia medications, the chest pain exhibited improvement. Coronary angiography revealed a true bifurcation lesion in the left main trunk (classified as Medina 1.1.1 type, with stenosis percentages of 80%, 85%, and 80%) [4] (Fig. 2). The IVUS examination revealed significant negative remodeling of the main trunk and branches at the distal bifurcation of the left main coronary artery, characterized by a lower proportion of atherosclerotic plaque components (Fig. 3). Supplementary Materials include coronary angiography films and IVUS films. In light of these findings, it was recommended to opt for coronary artery bypass graft surgery instead of performing coronary intervention with stent implantation. However, considering that coronary artery lesions are likely a consequence of radiation exposure, the implementation of medical treatment effectively alleviated symptoms of angina pectoris, thereby prompting the adoption of an internal medicine approach.

## Discussion

Radiation-induced cardiac injury is occasionally observed in malignant tumors such as mediastinal tumors, lymphomas, lung cancer, esophageal tumors, and breast tumors. It primarily presents as radiation pericarditis, pericardial effusion, heart failure, coronary artery disease, valvular heart disease, and arrhythmias. As the radiation dosage increases, there is a corresponding escalation in the risk of developing cardiovascular diseases. In a series of studies investigating adjuvant radiotherapy for breast cancer patients, it was observed that left-sided breast cancer patients receiving radiotherapy exhibited an 18% elevated risk of myocardial ischemia compared to their right-sided counterparts [5], suggesting that radiation therapy in close proximity to cardiac organs results in more significant cardiac damage [6]. Vascular damage induced by radiotherapy has been consistently observed in various studies; however, limited knowledge exists regarding its impact on the coronary arteries. Most studies indicate that radiation-induced injury to the coronary arteries is characterized by the development of atherosclerosis, initiated by damage to endothelial cells and subsequent infiltration of monocytes into the intima, resulting in deposition of low-density lipoprotein and formation of lipid streaks. As an exogenous influencing factor, radiotherapy has the potential to induce damage to endothelial cells, accelerate the progression of atherosclerosis in high-risk patients, and contribute to clinical events. Clinical studies have reported that the incidence of coronary heart disease among patients receiving adjuvant radiotherapy can be as high as 85%, which is closely associated with factors such as radiation dosage, location, and duration [7]. According to a study conducted by the Netherlands Cancer Institute, patients undergoing high-dose radiotherapy exhibited a cumulative incidence rate of 34.5% for coronary heart disease over a period of 25 years, thereby increasing their risk by 3–5 times compared to the general population [8]. In this case, the stenotic lesions of coronary artery were located in the irradiated field with relative high dose (Fig. 1C-E), which was also suggested the degree of coronary stenosis was related with the dose of radiotherapy. Moreover, oxidative stress has been implicated in inducing necrosis and fibrosis in the media and adventitia layers of coronary arteries [9]. Furthermore, case reports have indicated that tumor necrotic tissue may exert external compression on the coronary arteries of the heart, resulting in occlusion and diminished myocardial blood flow. This mechanism can precipitate ischemic cardiovascular conditions such as angina pectoris and myocardial infarction [10].

Importantly, previous studies have not reported any instances of chemoradiotherapy causing negative remodeling of the coronary arteries and inducing NSTEMI in patients with thymic carcinoma who underwent curative resection surgery and chemoradiotherapy. Radiation-induced cardiac toxicity has been implicated in the development and progression of various cardiovascular complications, including coronary artery disease. Several studies have documented the occurrence of radiation-induced coronary artery stenosis and plaque formation [11, 12]. However, there is a lack of comprehensive multimodality imaging approaches to assess the specific characteristics of radiation-induced coronary artery lesions, particularly in patients with thymic carcinoma who have undergone curative resection surgery and chemoradiotherapy. In this case, the patient was diagnosed with thymoma, and surgical exploration revealed cancer tissue infiltration into both sides of the pericardium and upper lobe of the right lung, encircling the superior vena cava and adjacent to cardiac vessels. Following surgical resection and subsequent chemotherapy due to recurrence, radiation therapy was administered resulting in notable improvement. Subsequently, the patient was readmitted due to intermittent chest tightness and pain. The patient exhibited significant coronary artery changes in the absence of conventional risk factors and without any prior evidence of cardiovascular disease. Angiography showed a true bifurcation lesion in the left main stem with less plaque burden at bifurcation site as indicated by IVUS examination. The coronary artery were contoured by later supplementation, and the stenotic area of coronary artery were located in the irradiated field with relative high dose. The observed changes, including augmented intima-media thickness and localized concentric narrowing fibrosis in narrow segments, have not been extensively elucidated within the context of radiation-induced coronary heart disease. Notably, this case highlights the presence of flat plaques in the affected coronary arteries, which is a notable departure from the typical plaque morphology seen in atherosclerotic coronary atherosclerotic disease. The absence of significant plaque calcification or ulceration further distinguishes the radiation-induced vascular changes from conventional atherosclerotic plaque development and progression. Concurrently, our data suggests that chemoradiotherapy may induce endothelial cell damage, leading to an inflammatory response and resulting in significant negative remodeling primarily through vessel constriction at the sites of opening with reduced plaque burden. Furthermore, this observation emphasizes the potential role of chemoradiotherapy as an potential independent risk factor for NSTEMI development, even among patients with an otherwise low-risk profile.

Conventional stent implantation may result in vascular rupture at sites of negative remodeling, leading to increased complications and mortality rates during coronary intervention procedures. Therefore, it is recommended that patients with a history of chest or mediastinal tumor chemoradiotherapy undergo routine intravascular imaging examinations prior to coronary intervention treatment in order to ascertain their specific characteristics and facilitate the adoption of appropriate methods. Therefore, we employed a systematic and integrated approach utilizing coronary angiography and IVUS to evaluate the coronary artery plaque characteristics in a patient with thymic carcinoma treated with surgery and chemoradiotherapy. This comprehensive imaging approach allowed for a detailed assessment of the plaque morphology, composition, and distribution, providing valuable insights into the nature of radiation-induced coronary artery changes in this specific clinical context. However, it is worth noting that in this particular case, the patient underwent not only radiation therapy but also chemotherapy, both of which have the potential to induce damage to the coronary arteries. Therefore, it can be inferred that the occurrence of coronary artery injury in this patient could be attributed to a combination of multiple factors, including the administration of radiation therapy and chemotherapy. Nonetheless, additional studies with larger sample size are warranted to explore these observations further.

Kouerinis IA also documented a case of emergency coronary artery bypass graft surgery in a patient with prior radiotherapy for thymic carcinoma [13]. However, there is currently a dearth of relevant series of studies investigating whether the incidence rate of restenosis within stents or graft occlusion increases in patients with a history of chest radiotherapy who undergo stent implantation or coronary artery bypass grafting due to the oxidative stress and inflammatory reactions induced by radiation therapy. Further large-scale observational studies are warranted to explore the imaging characteristics associated with radiation-induced damage. This underscores the necessity for advancing and refining novel radiation therapy techniques to minimize normal tissue exposure, achieve higher tumor irradiation doses, and enhance protection for normal tissues. Radiobiology and radiation therapy technology should be further explored and perfected [14, 15].

### Electronic supplementary material

Below is the link to the electronic supplementary material.

Supplementary Material 1

Supplementary Material 2

Supplementary Material 3

Supplementary Material 4

Supplementary Material 5

Supplementary Material 6

Supplementary Material 7

Supplementary Material 8

## Data Availability

The datasets used and/or analysed during the current study available from the corresponding author on reasonable request.
